# HIF1α-Dependent Induction of *TFRC* by a Combination of Intestinal Inflammation and Systemic Iron Deficiency in Inflammatory Bowel Disease

**DOI:** 10.3389/fphys.2022.889091

**Published:** 2022-06-08

**Authors:** Raphael R. Fagundes, Arno R. Bourgonje, Shixian Hu, Ruggero Barbieri, Bernadien H. Jansen, Nienke Sinnema, Tjasso Blokzijl, Cormac T. Taylor, Rinse K. Weersma, Klaas Nico Faber, Gerard Dijkstra

**Affiliations:** ^1^ Department of Gastroenterology and Hepatology, University of Groningen, University Medical Center Groningen, Groningen, Netherlands; ^2^ School of Medicine and Medical Science and the Conway Institute, University College Dublin, Dublin, Ireland

**Keywords:** HIF1α, TFRC, iron deficiency, inflammation, inflammatory bowel disease

## Abstract

**Background and Aims:** Iron deficiency (ID) is a frequent extra-intestinal manifestation in patients with Inflammatory Bowel Disease (IBD), who often do not respond to iron supplementation. Iron is a cofactor for hydroxylases that suppress the hypoxia-inducible factor-1α (HIF1α), a transcription factor regulating iron homeostasis. We hypothesized that iron deficiency affects mucosal HIF1α activity in IBD.

**Methods:** IBD patients (*n* = 101) were subdivided based on iron status (ferritin levels or transferrin saturation) and systemic inflammation (C-reactive protein levels). 154 corresponding ileal and colonic biopsies were analyzed for differential expression of 20 HIF1α pathway-associated genes and related to iron and inflammation status. *In vitro* expression of selected HIF1α pathway genes were analyzed in wild-type and *HIF1A-*null Caco-2 cells.

**Results:** Gene expression of the mucosal HIF1α pathway was most affected by intestinal location and inflammatory status. Especially, ileal mucosal *TFRC* expression, encoding the transferrin receptor TFR1, was increased in inflamed tissue (*p* < 0.001), and further enhanced in ID. Accordingly, *TFRC* expression in inflamed tissue associated negatively with serum iron levels, which was not observed in the non-inflamed mucosa. The HIF1α pathway agonist DMOG increased *TFRC* expression in Caco-2 cells, which was blunted in *HIF1A*-null cells.

**Conclusion:** We demonstrate that inflammation and anatomical location primarily determine HIF1α pathway activation and downstream *TFRC* expression in the intestinal mucosa. IBD patients with ID may benefit from treatment with HIF1α-agonists by 1) increasing *TFRC*-mediated iron absorption in non-inflamed tissue and 2) decreasing mucosal inflammation, thereby improving their responsiveness to oral iron supplementation.

## Introduction

Impaired iron homeostasis frequently results in anemia, which is a common co-morbidity of inflammatory diseases. In particular, patients with inflammatory bowel disease (IBD) develop anemia at a high rate, varying from 20%–40%, and it is considered an extra-intestinal manifestation of the disease leading to symptoms such as fatigue, weakness and dizziness, and a reduced quality of life (QoL) (Voegtlin et al., 2010; Bager et al., 2011; Ott and Scholmerich, 2013; Filmann et al., 2014; Herrera-Deguise et al., 2016; González Alayón et al., 2018). Anemia is a late manifestation of iron deficiency, which is an underdiagnosed and undertreated condition in IBD (Peyrin-Biroulet et al., 2018; Jimenez and Gasche, 2019). According to the European Crohn’s and Colitis Organization (ECCO) guidelines, intravenous iron should be the first-line treatment in patients with active disease (Dignass et al., 2015). Both in patients with active and quiescent disease, it remains difficult to predict patient responsiveness to oral iron supplementation. Furthermore, other therapeutic options, such as erythropoiesis-stimulating agents and blood transfusions, are limited (Murawska et al., 2016; Patel et al., 2018). Anemia and disease progression in IBD are linked, since aggravation of IBD promotes factors that lead to iron deficiency (ID) anemia. As a consequence, iron-deficient patients develop impaired immune function and thromboregulation, hair loss, glossitis, and fatigue. The latter represents the biggest burden, which significantly impairs QoL of these patients (Pizzi et al., 2006; Herrera-Deguise et al., 2016). The etiology of ID in IBD is not fully understood, however, intestinal blood loss, low intake, and decreased hepcidin-regulated absorption of iron are known factors to contribute to reduced iron levels (Kaitha et al., 2015).

Iron participates in numerous biochemical functions in our body, including oxygen transport, energy production, immune regulation, and response to hypoxia (Lawen and Lane, 2013). Intracellular iron has a direct effect on regulating the transcriptional activity of the hypoxia-inducible factor (HIF) pathway. The HIF pathway is composed of two subunits: an alpha (*α*) subunit, directly regulated by oxygen levels, and a constitutively expressed beta (*β*) subunit (Wang et al., 1995). There are three HIFα isoforms described in mammalian cells: HIF1α, HIF2α, and HIF3α, all presenting a different range of gene targets (Schito and Semenza, 2016), while the latter remains not yet well-described in literature. Altogether, iron is a critical cofactor of hydroxylases that target the HIFα subunit for degradation in the presence of oxygen (normoxia), whereas a state of hypoxia or decreased iron availability blocks this reaction and HIF1α becomes transcriptionally active. The hydroxylase reaction is controlled by prolyl-hydroxylases (EGLN1, 2, and 3) and asparaginyl-hydroxylase Factor inhibiting HIF1 (FIH-1). Of special importance, HIF1α and HIF2α-pathway activation leads to downstream activation of a number of target genes that, besides various other cellular pathways, collectively participate in systemic and cellular iron homeostasis (Shah and Xie, 2014; Hirota, 2019). Relevant genes include hepcidin (*HAMP*), heme oxygenase-1 (*HMOX1*), aconitase (*IRP1*) and erythropoietin (*EPO*). Importantly, HIF1α activation modulates both extra- and intracellular iron levels by regulating the expression of the transport protein transferrin (TF, encoded by *TF* gene), and cell surface transferrin receptor 1 protein (TFR1, encoded by the *TFRC* gene) (Cheng et al., 2015; Muckenthaler et al., 2017; Chen et al., 2020; Noonan et al., 2021).

The intestinal mucosa has a physiologic hypoxia gradient along the crypt-villous axis (Zheng et al., 2015), and active inflammation leads to a more severe and extensive state of pathophysiologic hypoxia (Brown and Taylor, 2018). The HIF1α pathway is a key player in maintaining gut homeostasis, by modulating epithelial barrier and immune cell function (Fagundes and Taylor, 2017; Taylor and Colgan, 2017). In addition, HIF1α is an attractive target for improving the systemic iron status of patients with ID, since HIF1α pathway activation targets genes involved in the promotion of blood oxygenation and iron homeostasis, particularly *TF* and *TFRC*. Accordingly, an interplay between iron levels and the HIF1α pathway may be a crucial mechanism underlying ID in IBD patients. In this study, we aimed to gain more insight into this interplay by correlating serum iron status and activation of the HIF1α pathway and targeted genes in matched mucosal biopsies of IBD patients.

## Materials and Methods

### Study Population

All patients included in this study were recruited from the IBD center of the of the Department of Gastroenterology and Hepatology of the University Medical Center Groningen (UMCG) and consented to participate in the 1000IBD project and Dutch Parelsnoer IBD Biobank (Spekhorst et al., 2017; Imhann et al., 2019). Patients were at least 18 years old and had an established diagnosis of IBD existing for at least 1 year. Diagnosis was based upon clinical, laboratory, endoscopic, and histopathological criteria, the latter criteria also used for determining the inflammatory status of collected tissues (Lennard-Jones, 1989). Inclusion was further based on the availability of intestinal biopsies for the generation of transcriptome data and serum laboratory data retrieved at less than 30 days to the biopsy collection date.

### Study Design and Data Collection

**FIGURE 1:**
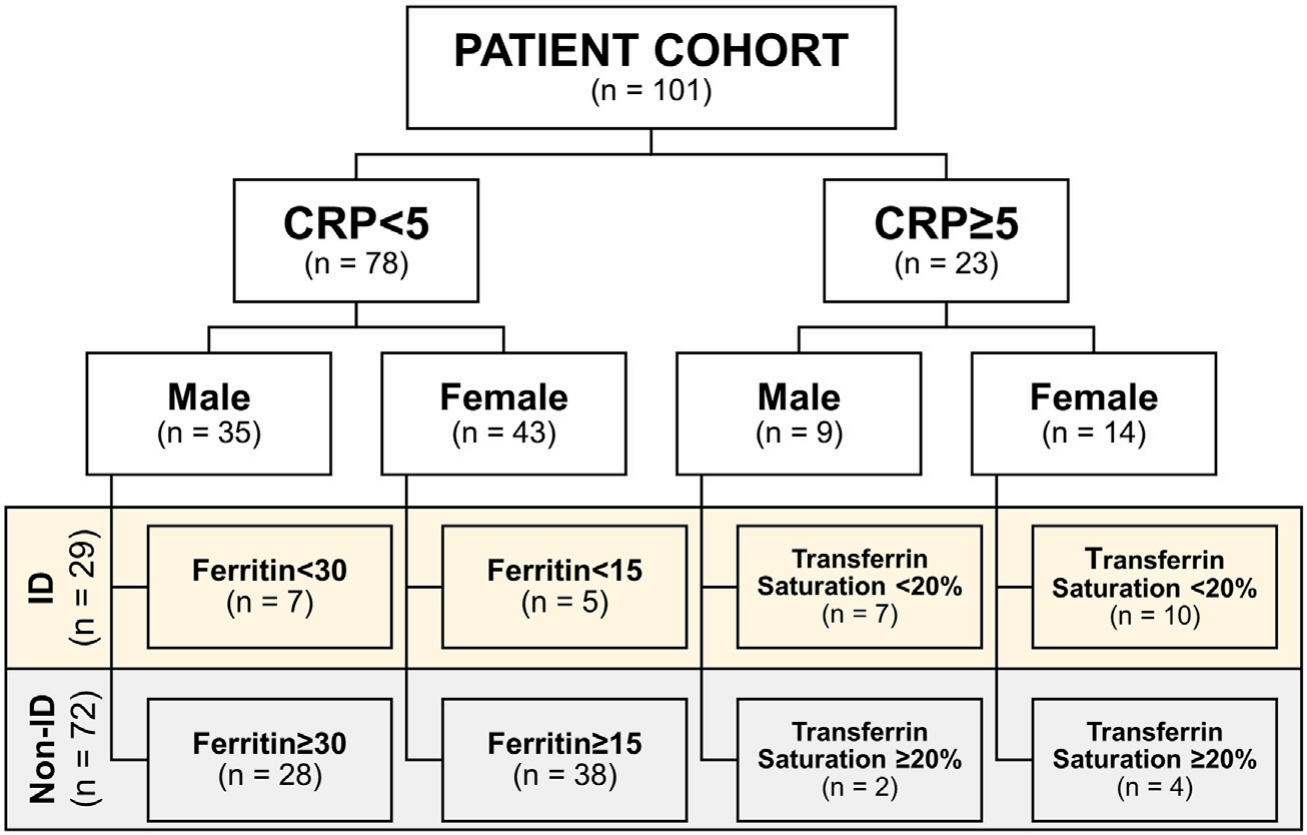
Cohort subdivision in iron deficiency (ID) and non-ID groups according to criteria. CRP, C-reactive protein.

Upon study inclusion, demographic, and clinical characteristics of the study population were collected, including age, sex, body-mass index (BMI, body weight divided by squared height), medication usage, smoking behavior, and disease location according to the Montreal classification. Serum laboratory blood parameters were recorded, including hemoglobin, mean corpuscular cell volume (MCV), C-reactive protein (CRP), as well as iron status parameters, including free iron, ferritin, total iron binding capacity (TIBC), transferrin, and transferrin saturation (Roche Modular, Roche, Mannheim, and Germany). The definition of iron deficiency (ID) was based on serum ferritin levels in case of normal serum CRP (<5 mg/L; ferritin <30 μg/L for males or <15 μg/L for females) or based on the transferrin saturation in case of elevated CRP levels (≥5 mg/L; transferrin saturation < 20%), as ferritin is also an acute-phase reactant and sensitive to systemic inflammation (Bermejo and García-López, 2009; Mowat et al., 2011; Lupu et al., 2015; Cappellini et al., 2017). Subsequently, patients were categorized in this study as having “normal iron status” or “iron deficient” (Figure 1; non-ID and ID, respectively).

### Immunohistochemistry of Human Intestinal Material

Intestinal material was obtained from patients with IBD undergoing surgical resection. Tissue was collected from macroscopically inflamed areas and flanking (non-inflamed) regions. Intestinal tissue was stored in an ice-cold University of Wisconsin (UW) tissue preservation solution until further use. Immunohistochemistry was performed as described previously (Trentadue et al., 2020). Briefly, paraffin-embedded tissue sections of 4 μm from intestinal tissue from resection material were cut and placed on Starfrost slides (3054-1, Klinipath, VWR, Breda, Netherlands). Sections were dried, deparaffinized in xylene, and rehydrated in alcohol, prior to blockage of endogenous peroxidase with 0.3% H_2_O_2_ in phosphate-buffered solution (PBS) for 30 min. The slides were then blocked for 30 min with 1% bovine serum albumin (BSA)/PBS before being incubated for 1 h at room temperature with a primary antibody against TfR1 (1:200, catalog no. 84036, Abcam, Cambridge, United Kingdom). The secondary and tertiary steps were performed for 3 min with horseradish peroxidase-labeled antibodies (1:50, Dako, Agilent, Santa Clara, CA, United States; rabbit antimouse and goat antirabbit, respectively) in 1% BSA/PBS supplemented with 1% human serum. Binding of anti-TfR1 antibody was detected by 3,3-diaminobenzidine and counterstained with hematoxylin.

### RNA Sequencing of Intestinal Biopsies

Intestinal biopsies were collected within the context of the 1000IBD project and Dutch IBD Biobank at time of colonoscopy procedures, which were part of standard clinical care. Intestinal biopsies were readily snap-frozen in liquid nitrogen shortly after the colonoscopy procedure. In total, 154 intestinal biopsies from 101 patients with IBD were analyzed in this study. Biopsy inflammatory status was macroscopically classified during the endoscopy procedure based on the aspect of the mucosa. Macroscopic inflammation status was defined as redness and edema with or without ulceration of the intestinal mucosa. Furthermore, macroscopic inflammation status was later confirmed by histopathological evaluation. Biopsies were stored at −80°C until further processing. Isolation of RNA was performed using the AllPrep DNA/RNA mini kit (Qiagen; reference number: 80204) according to the manufacturer’s protocol. Homogenization of intestinal biopsies was performed in RLT lysis buffer including β-mercaptoethanol using the Qiagen Tissue Lyser with stainless steel beads (with a diameter of 5 mm; reference number: 69989). Sample preparation was performed using the BioScientific NextFlex mRNA sample preparation kit. RNA sequencing was executed with the Illumina NextSeq500 sequencer. Sampling and sequencing were performed in two different batches. Therefore, RNA samples were pseudo-randomized on plates to mitigate potential batch effects. However, no relevant batch effect has been determined by means of principal coordinate analyses that were conducted as part of the quality control. At least 20 million reads were approximately generated per sample.

The quality of the raw reads was checked using FastQC at default parameters (ref v0.11.7). The adaptors identified by FastQC were clipped using Cutadapt (ref v1.1) with default settings. Sickle (ref v1.200) was used to trim low-quality ends from the reads (length <25 nucleotides, quality <20). Reads were aligned to the human genome (human_g1k_v37) using HISAT (ref v0.1.6) (2 mismatches allowed) and read sorting was done by SAMtools (ref v0.1.19). SAMtools flagstat and Picard tools (ref v2.9.0) were used to obtain mapping statistics. Seven samples with low percentage read alignment (<90%) were removed. Finally, the gene expression was estimated through HTSeq (ref 0.9.1) based on Ensemble version 75 annotation, resulting in an RNA expression dataset featuring 28,716 genes.

From the RNA sequencing data, 20 different HIF pathway related genes were selected for analysis. Genes that were analyzed included *HIF1A*, *HIF2A*, HIF-hydroxylases (*EGLN1*, *EGLN2*, and *EGLN3*), *VHL*, *HIF1AN*, and HIF1α/-2α target genes (*CA9*, *PDK1*, *SLC2A1*, *MUC3A*, *TFF3*, *A2BAR*, *SLC29A1*, *ADK*, *HAMP*, *SLC11A2*, *HMOX1*, *TFRC*, and *TF*). In addition, HIF1α and HIF2α scores were calculating as described by Brown et al. (2020).

### CRISPR/Cas9 Genetic Editing, Cell Culture and Treatments

Human epithelial colon adenocarcinoma cells (Caco-2, male, ATCC^®^, HTB-37TM, Manassas, VA, United States) were used as *in vitro* representatives of intestinal epithelial cells. Single guide RNA’s for *HIF1A* (sequence 5′-GAT​GGT​AAG​CCT​CAT​CAC​AG-3′) were designed *via* Benchling^©^ online platform (https://www.benchling.com/) and cloned in pLenti-sgRNA backbone (Addgene, 71409). For lentivirus production, HEK293T cells in T25 were transfected with Fugene HD (Promega, E2311), 250 μl Opti-MEM, 15 μl Fugene, 1.5 μg Lenti-iCas9-neo (Addgene, 85400) or pLenti-sgRNA (see above), 1 μg pMD2. G (Addgene, 12259) and 2.5 μg psPAX2 (Addgene 12260). Medium was washed 24 h after transfection and changed for Glutamax™ Dulbecco’s Modified Eagle Medium (DMEM, ThermoFisher Scientific Inc.), supplemented with 10% fetal calf serum (FCS, Invitrogen), 1% non-essential amino acids (NEAA, Gibco) and 1% PSF antibiotic cocktail [penicillin (10 U/ml), streptomycin sulfate (100 μg/ml) and fungi zone; Lonza, Basel, Switzerland]. Lentivirus suspension was collected 48 h after transfection, filtered through a 0.45 μm filter, aliquoted and stored at −80°C.

For generating stable Caco-2-*HIF1A*-null cell line, Caco-2 cells were seeded in 6-well plate and incubated with 1 ml lentivirus suspension, supplemented with 8 μg/ml polybrene (Sigma-Aldrich, TR-1003). Cells were washed 24 h after infection with PBS and selection was started with Neomycin 2 mg/ml for 7 days with one passage in between. For introducing sgRNA, Caco-2 cells were seeded in 6-well plate and incubated with 1 ml lentivirus containing sgRNA (empty vector or against *HIF1A*—described above), supplemented with 8 μg/ml polybrene. Cells were washed 24 h after infection with PBS and selection was started 20 μg/ml puromycin for 7 days. Once puromycin treatment was finished, the resulting mutant Caco-2 cell line was cultured in low density on Glutamax™ DMEM, as described above. 21 clones were picked from culture dish and cultured individually. Clones were expanded and DNA, RNA and protein collected for sequencing and functional validation of transfection. Prior to experiments, cells were seeded to at least 80% confluence on 12-well plates. Treatments with control or Dimethyloxalylglycine (DMOG; D3695, Sigma Aldrich), at the concentrations of 1 or 5 mM, were carried out for 24 h, followed by RNA isolation.

### RNA Isolation and Quantitative RT-PCR

RNA was isolated from Caco-2 cells using TRIzol (Sigma-Aldrich) according to the manufacturer’s instructions, followed by quantification of RNA using NanoDrop 2000c spectrophotometer (Thermo-Scientific). Subsequently, complementary DNA (cDNA) synthesis using reverse transcriptase PCR was performed. To a final volume of 50 μl, we added 2.5 ng of RNA from each sample, 10% reaction RT Buffer (final concentration 50 mM Tris-HCl, 50 mM KCl, 3 mM MgCl_2_, and 5 mM DTT), 10% dNTP mix (final concentration 1 mM dATP, dGTP, dTTP, Sigma-Aldrich), 2% random primers (0.01 μg/μl; Sigma-Aldrich), 2% M-MLV RT (100 U in 50 μl final volume; Invitrogen) and 1.5% RNAse OUT (30 U in 50 μl final volume; Invitrogen). cDNA synthesis was performed on a thermal cycler (Bio-Rad T100) for 10 min at 25°C followed by 60 min at 37°C and 5 min at 95°C. Prior to quantitative real-time PCR, cDNA solution for each sample was diluted 20-fold in RNAse-free water.

Gene expression of *HIF1A* and transferrin receptor *TFRC* were measured by TaqMan-based quantitative Real-Time PCR (RT-PCR). Housekeeping ribosomal *18S* was used for normalization. Supplementary Table S3 presents the sequence and description of the probes and primers used in this study. Each sample was prepared in duplicates, in a reaction mix (final volume 20 μl) containing 0.2 μM fluorescent probes, 0.936 μM forward and reverse primers, 10 μl qPCR reaction buffer (Eurogenic), 4.48 μl RNAase-free water and 4 μl cDNA. Amplification was allowed by 10 min heating at 95°C followed by a 40-times repeated cycle, consisting of 15 s at 95°C and 1 min at 60°C, performed using a StepOnePlus (AB, Applied Biosystems) PCR system.

### Statistical Analysis

Data were presented as mean ± standard deviation (SD), medians with interquartile ranges (IQR) in case of skewed distributions or proportions *n* with corresponding percentages (%). Assessment of normality was performed using histograms and normal probability plots (Q-Q plots). Continuous variables were compared between groups using independent sample *t*-tests or Mann-Whitney *U*-tests, while chi-square tests or Fisher’s exact tests were used to compare for comparison of categorical variables. Gene expression was normalized using the trimmed mean of M-values normalization method (TMM) and then ^2^log-transformation was applied. Finally, gene expression means were centered to zero and standard deviations scaled to one.

Principal component analyses (PCA) and variation expression analyses were conducted to determine which variables contributed most to the variation in gene expression. To assess the contributing effect of each factor on total gene expression, the Adonis function was used to perform ANOVA with 1,000 permutations. Comparative analyses for gene expression were performed using a linear mixed model, expressed by the following formula:

Gene expression level∼IBD diagnosis+iron status+CRP level+tissue inflammatory status+tissue location+age+sex+medication usage+(1|repeated measurements)

The term “repeated measurements” was incorporated in the model as a random effect to control for potential bias due to the inclusion of multiple biopsies for selected patients. Data were analyzed using SPSS Statistics 25.0 software package (SPSS Inc., Chicago, IL, United States) and R version 3.5.2 (Vienna, Austria). Data visualization was performed using GraphPad Prism 9.0 (GraphPad software, San Diego, CA, United States) and RStudio (version 1.2.1335; RStudio, Boston, MA, United States). Two-tailed *p*-values ≤ 0.05 were considered statistically significant.

### Ethical Considerations

All patients included in this study were asked to provide written informed consent to participate. The study was approved by the Institutional Review Board (IRB) of the University Medical Center Groningen (UMC Groningen) (in Dutch: “Medisch Ethische Toetsingscommissie,” METc; IRB no. 2008/338 and 2016/424). The study was performed in accordance with the principles of the Declaration of Helsinki (2013).

## Results

### Baseline Characteristics of the Study Population

The study population is composed of 101 IBD patients that had laboratory results available at the closest dates to biopsy collection for RNA sequencing [median (IQR) time interval 1.3 (0.7–3.0) months; Figure 1]. Twenty-nine (29) patients classified as ID and 72 as non-ID (Table 1; ID criteria described in *Materials and Methods*). IBD-ID patients presented a higher use of corticosteroids, compared to non-ID patients (*p* = 0.032). Table 2 shows values for laboratory iron status parameters between non-ID and ID patients. ID patients presented lower iron status parameters, i.e., significantly lower values of MCV, free iron, ferritin and transferrin saturation, providing a validation for the group subdivisions.

**TABLE 1:** Descriptive statistics of IBD patients with differences shown between IBD patients with normal iron status (non-ID) and iron deficiency (ID).

Variable	Non-ID	ID	*p*-value
	*n* = 72	*n* = 29	
Age (years)	45.1 ± 15.0	43.4 ± 14.6	0.625
Female gender, n (%)	42 (58.3)	15 (51.7)	0.544
BMI (kg/m^2^)	25.0 ± 4.1	25.8 ± 4.0	0.364
Current smoking, n (%)	16 (22.2)	3 (10.3)	0.167
IBD type			0.437
CD, *n* (%)	41 (56.9)	20 (69.0)	
UC, *n* (%)	27 (37.5)	7 (24.1)	
IBD-U, *n* (%)	4 (5.6)	2 (6.9)	
Montreal classification (Age)			0.697
A1 (≤16 years), *n* (%)	8 (11.4)	2 (7.1)	
A2 (17–40 years), *n* (%)	42 (60.0)	16 (57.1)	
A3 (>40 years), *n* (%)	20 (28.6)	10 (35.7)	
Montreal classification (Location) (CD)			0.108
L1 (ileal disease), *n* (%)	13 (31.0)	2 (10.0)	
L2 (colonic disease), *n* (%)	8 (19.0)	4 (20.0)	
L3 (ileocolonic disease), *n* (%)	18 (42.9)	10 (50.0)	
L4 (upper GI disease), *n* (%)	0 (0.0)	1 (5.0)	
Montreal classification (Behavior) (CD)			0.350
B1 (non-stricturing, non-penetrating), *n* (%)	17 (40.5)	12 (60.0)	
B2 (stricturing), *n* (%)	18 (42.9)	6 (30.0)	
B3 (penetrating), *n* (%)	7 (16.7)	2 (10.0)	
Montreal classification (Perianal disease) (CD)			0.308
Yes, *n* (%)	16 (38.1)	5 (25.0)	
No, *n* (%)	26 (61.9)	15 (75.0)	
Montreal classification (Extension) (UC)			0.367
E1 (proctitis), *n* (%)	2 (7.4)	0 (0.0)	
E2 (left-sided colitis), *n* (%)	7 (25.9)	4 (50.0)	
E3 (pancolitis), *n* (%)	18 (66.7)	4 (50.0)	
Montreal classification (Severity) (UC)			0.682
S0 (remission), *n* (%)	1 (3.7)	0 (0.0)	
S1 (mild disease), *n* (%)	4 (14.8)	2 (25.0)	
S2 (moderate disease), *n* (%)	14 (51.9)	5 (62.5)	
S3 (severe disease), *n* (%)	8 (29.6)	1 (12.5)	
Concomitant medication use (IBD)			
Medication (any), *n* (%)	64 (88.9)	24 (82.8)	0.405
Aminosalicylates, *n* (%)	31 (43.1)	9 (31.0)	0.264
Thiopurines, *n* (%)	27 (37.5)	6 (20.7)	0.103
Corticosteroids, *n* (%)	21 (29.2)	15 (51.7)	0.032*
TNF-antagonists, *n* (%)	21 (29.2)	6 (20.7)	0.384
Methotrexate, *n* (%)	4 (5.6)	5 (17.2)	0.062

Data are presented as mean ± standard deviation (SD), median (interquartile range, IQR) or proportions n with corresponding percentages (%). BMI, body mass index; CD, Crohn’s disease; UC, ulcerative colitis; IBD-U, undetermined IBD. *p-value indicating statistical significance.

**TABLE 2:** Relevant laboratory parameters of IBD patients with differences shown between IBD patients with normal iron status (non-ID) and iron deficiency anemia (ID).

Variable	Non-ID	ID	*p*-value
	*n* = 72	*n* = 29	
Hemoglobin (mmol/L)	8.5 ± 1.0	8.2 ± 0.8	0.164
MCV (fL)	91.6 ± 6.1	87.7 ± 6.1	0.007*
*CRP (mg/L)*			<0.001*
<5 mg/L	66 (91.7)	12 (41.4)	
≥5 mg/L	6 (8.3)	17 (58.6)	
Free iron (µmol/L)	18.3 ± 7.2	9.5 ± 3.8	<0.001^*^
Ferritin (µg/L)^†^	63.5 [34.3; 126.5]	21.0 [12.8; 30.5]	<0.001^*^
TIBC (µmol/L)	63.9 ± 12.1	68.0 ± 14.8	0.235
Transferrin (g/L)	2.7 ± 0.5	3.0 ± 0.5	0.120
Transferrin saturation (%)^†^	26.7 [21.6; 35.7]	15.9 [9.4; 18.0]	<0.001^*^

Data are presented as mean ± standard deviation (SD), median (interquartile range, IQR) or proportions n with corresponding percentages (%). MCV, Mean corpuscular volume; CRP, C-reactive protein; TIBC, Total iron binding capacity. *p-value indicating statistical significance.

### Tissue Location and Inflammatory Status Contribute Most to the Variation in Expression of HIFα Pathway Genes

**FIGURE 2:**
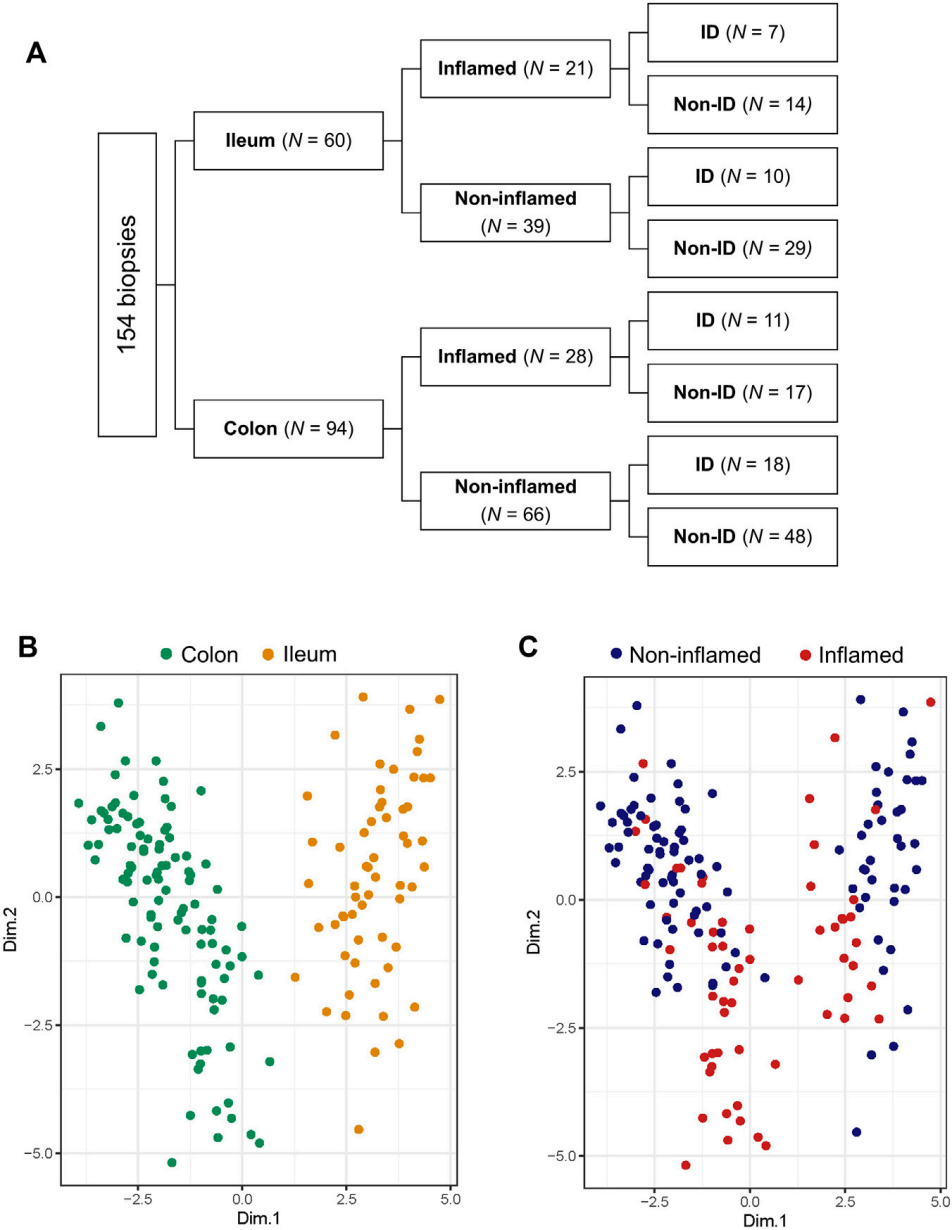
**(A–C)** Biopsy location and inflammatory status contribute the most to variation in gene expression. **(A)** Diagram showing subdivision of biopsies depending on location, inflammatory status and iron deficiency (ID) status and biopsy count per group analyzed by RNA sequencing. Principal component analysis (PCA) targeted for **(B)** biopsy location and **(C)** inflammatory status.

Of the 101 included patients, 154 intestinal biopsies (ileum: *n* = 60, colon: *n* = 94; non-inflamed: *n* = 105, inflamed *n* = 49) were available for analysis (Figure 2A). Based on relevant literature, twenty (20) HIF1α- HIF2α-related genes were selected from RNAseq data of intestinal biopsies of IBD patients, 13 of these HIF1- or HIF2-regulated genes (isoform-specific regulation and respective literature are indicated in Table 3). Principal component analysis (PCA) was performed on these 20 selected genes and distinct clusters were identified and expression levels of these genes were clearly differentiated by tissue location and inflammatory status (PC1 and PC2, respectively, Figures 2B,C; both *p* < 0.001). Based on this overall HIFα pathway characterization, expression data per sample were subdivided depending on anatomic location (ileum or colon), inflammatory status (based on histopathology), and systemic iron status (ID or non-ID) in subsequent analyses, as shown in Figure 2A.

**TABLE 3:** Description of analyzed HIF(1/2)-regulated genes.

Gene	Protein name	HIF	Function	References
*CA9*	Carbonic anhydrase 9	HIF1	Zinc metalloenzyme	Krieg et al. (2000), Kaluz et al. (2009), van den Beucken et al. (2009)
*PDK1*	Pyruvate dehydrogenase kinase	HIF1	Regulates metabolite flux through the tricarboxylic acid cycle	Kim et al. (2006)
*SLC2A1*	Glucose transporter 1	HIF1	Transmembrane transport of glucose	Chen et al. (2001), Hayashi et al. (2004)
*MUC3A*	Mucin 3	HIF1	Formation of mucus barrier	Louis et al. (2006)
*TFF3*	Trefoil factor 3 (or intestinal TFF)	HIF1	Formation of mucus barrier	Hernández et al. (2009)
*A2BAR* ^a^	A2B adenosine receptor	HIF1	Adenosin receptor	Eltzschig et al. (2003), Kong et al. (2006)
*SLC29A1* ^a^	Equilibrative nucleoside transporter 1	HIF1	Cellular uptake of nucleosides	Eltzschig et al. (2005)
*ADK*	Adenosine kinase	HIF1	Intracellular conversion of adenosine to adenosine-monophosphate	Elvidge et al. (2006), Morote-Garcia et al. (2008)
*HAMP* ^b^	Hepcidin	HIF2	Systemic iron homeostasis	Mastrogiannaki et al. (2009), Shah et al. (2009)
*SLC11A2* ^b^	Divalent metal transporter 1	HIF2	Cellular iron uptake	Mastrogiannaki et al. (2009), Shah et al. (2009)
*HMOX1* ^b^	Heme oxygenase-1	HIF1	Heme degradation	Lee et al. (1997)
*TFRC* ^b^	Transferrin receptor	HIF1	Transmembrane uptake of iron	Lok and Ponka (1999), Tacchini et al. (1999), Xu et al. (2017)
*TF* ^b^	Transferrin	HIF1	Serum transport of iron	Xia et al. (2009)

a Negatively regulated by HIF.

b genes encoding proteins involved in iron homeostasis.

### Ileal Tissue Presents Higher Sensitivity to HIFα Pathway Activation Compared to Colonic Tissue

**FIGURE 3:**
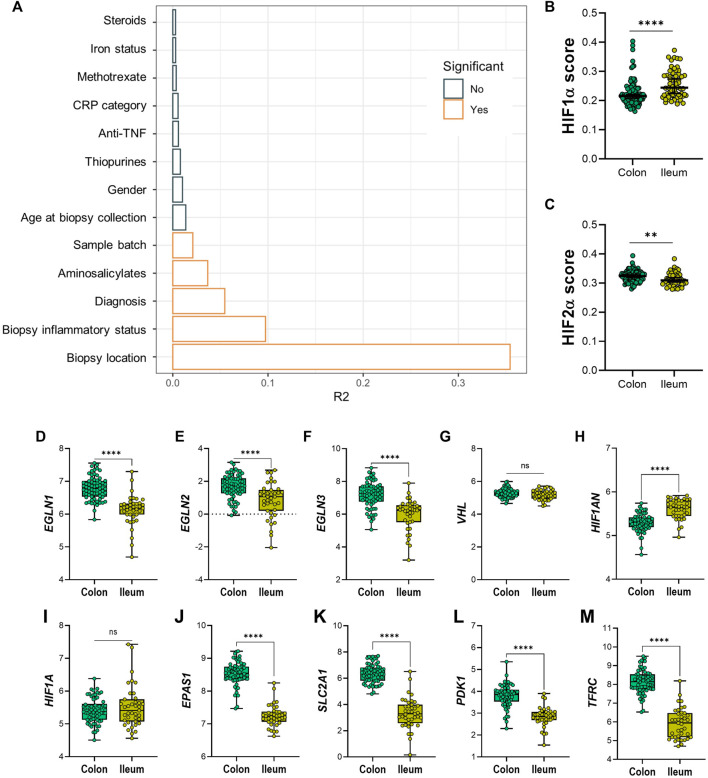
**(A–M)** Diagram **(A)** shows variables and effect on differential expression on dataset; significant confounders are highlighted in orange, as indicated by legend. **(B)** HIF1α and **(C)** HIF-2α scoring on colonic and ileal biopsies. Differential expression between non-inflamed colonic or ileal biopsies from IBD patients of genes involved in the HIF-α pathways; HIF-hydroxylases **(D)** *EGLN1*, **(E)** *EGLN2*, **(F)** *EGLN3*, **(G)** *VHL*, **(H)** *HIF1AN*, **(I)** *HIF1A*, and **(J)** *EPAS1*/HIF-2α; and HIF-α gene targets **(K)** *SLC2A1*, **(L)** *PDK1* and **(M)** *TFRC*. Data in figures **(B,C)** are presented as scattered dots with median with 95% CI. Data in figures **(D–M)** are presented as boxplots with minimum to maximum whiskers. ID, iron deficient; Non-ID, not-iron deficient; TNF, tumor necrosis factor; CRP, C-reactive protein. ^**^ *p* < 0.01; ^****^ *p* < 0.0001; ns, not significant.

Permutational multivariate ANOVA was used to calculate the contribution of various uncontrolled covariates in the dataset to gene expression levels of the 20 HIF-pathway genes. In line with results from PCA, this revealed that intestinal location contributed the most to variation in expression of HIF-pathway related genes, followed by tissue inflammatory status (Figure 3A). In addition, IBD subtype, use of aminosalicylates (at time of sample collection) and sample processing batch contributed significantly to the variation of HIF1α pathway expression in analyzed biopsies. Adjustment for these covariates was performed using a linear mixed model design (see *Materials and Methods*). In order to rule out the major effect of inflammation on the expression levels of the analyzed genes, we next concatenated gene expression levels of different components of the HIF1α and HIF2α pathways in non-inflamed tissue (Figures 3B–M). Analysis of HIF1α and HIF2α scores revealed that the HIF1α score was significantly increased in ileal tissue compared to colonic tissue (*p* < 0.001). The opposite was observed for the HIF2α score, which was significantly lower in ileal compared to colonic tissue (*p* = 0.001) (Figures 3B,C).

At the individual gene level, non-inflamed ileal tissue presented lower levels of HIF-hydroxylases *EGLN1*, *EGLN2*, and *EGLN3*, when compared to non-inflamed colonic tissue (Figures 3D–F, respectively). In contrast, *VHL* expression levels were unchanged, while *HIF1AN* levels were significantly higher in ileum, compared to colon (Figures 3G,H). Expression levels of *HIF1A* were comparable, whereas *EPAS1* (encoding HIF2α) levels were significantly lower in ileal tissue, compared to colonic tissue (Figures 3I,J). Surprisingly, mRNA levels of HIF1α-targets were higher in colonic compared to ileal mucosa (Figures 3K–M).

### Inflammation and ID Modulate HIFα Pathway and *TFRC* Expression in Ileal Mucosa

**FIGURE 4:**
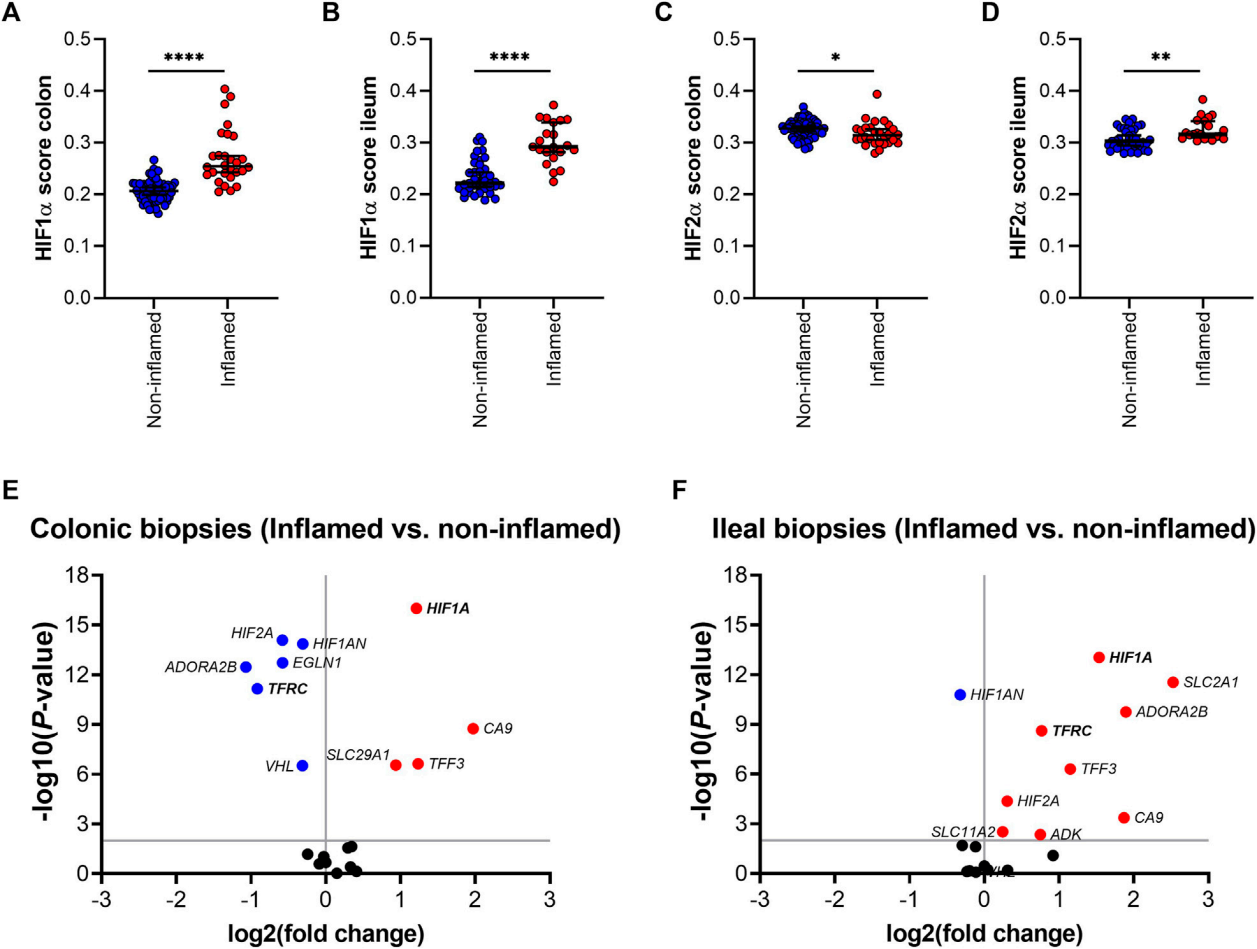
**(A–F)**; HIF1α **(A,B)** and HIF-2α **(C,D)** scoring in inflamed and non-inflamed biopsies from colon or ileum of IBD patients. Differential mRNA expression of genes involved in the HIF1α pathway in inflamed and non-inflamed biopsies (represented by volcano plots) in **(E)** colonic and **(F)** ileal biopsies. All data are presented as medians with 95% CI. ^*^ *p* < 0.05; ^**^ *p* < 0.01; ^****^ *p* < 0.0001.

Next, the effect of inflammation was analyzed on gene expression of HIFα pathway components in ileal and colonic biopsies. HIF1α and HIF2α scores were significantly increased in inflamed, compared to non-inflamed ileal mucosa (Figures 4B,D). HIF1α score was also increased in inflamed, compared to non-inflamed, colonic tissue, whereas the corresponding HIF2α score was reduced by inflammation (Figures 4A,C, respectively). Differential gene expression analysis is presented as volcano plots, in order to study the impact of mucosal inflammation on the expression of individual HIFα related genes (Figures 4E,F). We observed an inflammation-driven induction of components of the HIFα pathway that was more pronounced in ileal tissue, compared to colonic biopsies, especially for HIF1α downstream target genes. This aligns with the higher HIF1α scores in non-inflamed ileal compared to colonic mucosa (Figure 3B), which indicates an increased HIF1α activation capacity in the ileal mucosa. Interestingly, ileal *TFRC* expression was the top significant association among the five genes that are regulated by HIF1α and are involved in iron homeostasis (Figure 4F).

**FIGURE 5:**
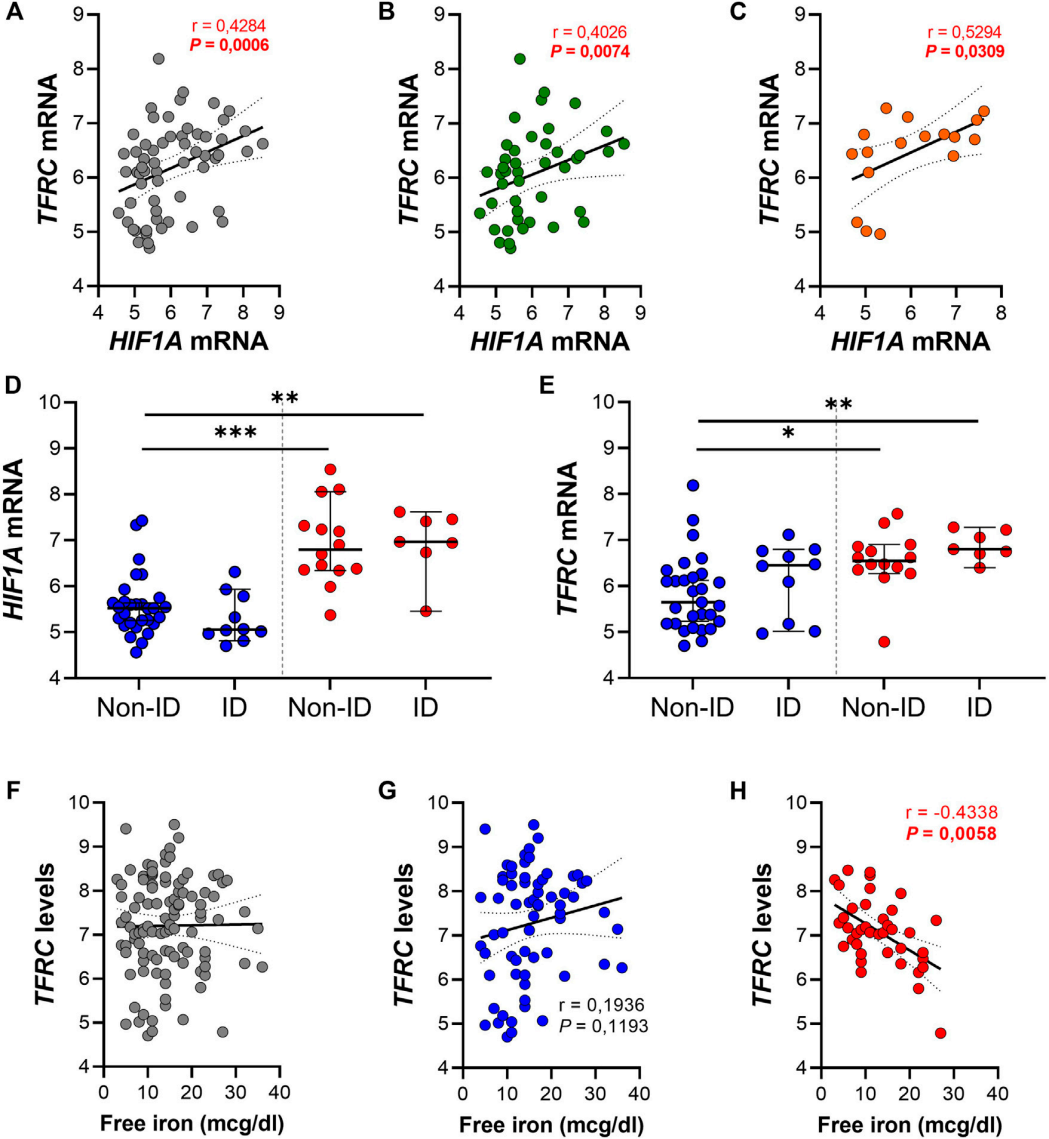
**(A–H)** Spearman correlation analysis between gene expression levels of *TFRC* and *HIF1A* in **(A)** ileal biopsies and ileal biopsies from **(B)** IBD non-ID and **(C)** IBD-ID patients. Differential gene expression of **(D)** *HIF1A* and **(E)** *TFRC* in ileal biopsies from non-inflamed (blue dots) and inflamed (red dots) areas, according to iron deficiency status of IBD patients at moment of biopsy collection. *TFRC* gene expression levels associated, by Spearman correlation, with serum iron levels and associations are shown with serum free iron levels in **(F)** the complete dataset and subdivided for **(G)** non-inflamed and **(H)** inflamed biopsies. Data presented in correlation graphs are presented as best fitting line with 95% confidence intervals. Data in figures **(D,E)** are presented as median with 95% CI. ID, iron deficiency. ^*^ *p* < 0.05; ^**^ *p* < 0.01; ^***^ *p* < 0.001.

In order to evaluate a potential association between the HIF1α pathway, ID, iron levels and *TFRC* gene expression, gene expression levels of both genes were further analyzed in ileal tissue (Figure 5). We found a significant positive correlation between ileal *HIF1A* and *TFRC* expression levels in ileal biopsies (*r* = 0.43 and *p* < 0.001; Figure 5A). This significant association remained present when ileal biopsies were stratified by systemic iron status, i.e., ileal tissue from IBD-non-ID and IBD-ID patients (Figures 5B,C, respectively). On the contrary, this significance was abrogated when stratifying tissue samples by inflammatory status (Supplementary Figure S1). To further dissect this association, we performed differential gene expression analysis for *HIF1A* and *TFRC* among ileal tissue segregated by the following: non-inflamed-non-ID; non-inflamed-ID; inflamed-non-ID; and inflamed-ID (Figures 5D,E). Here, *HIF1A* gene expression was enhanced in inflamed tissue (both non-ID and ID), compared to non-inflamed-non-ID tissue (Figure 5D). Similarly, *TFRC* gene expression was enhanced in inflamed ileal tissue, both in non-ID and ID patients. Interestingly, *TFRC* gene expression was more significantly upregulated in inflamed ileal tissue of ID patients compared to ileal tissue from non-ID patients (*p* = 0.019; Figure 5E). This comprehensive analysis pointed to a potential *TFRC*-HIF1α crosstalk in ileum that is highly dependent on the systemic iron status and revealed a cumulative effect of systemic ID and, especially, mucosal inflammation on the transcriptional regulation of ileal *TFRC* gene expression.

Lastly, we evaluated the impact of serum free iron (mcg/dl) on the transcriptional levels of mucosal *TFRC*. By performing Spearman correlation analysis between mucosal *TFRC* gene expression levels and serum free iron levels of the same patients, we observed that intestinal mucosal *TFRC* expression was not significantly associated with systemic free iron levels (*r* = 0.038 and *p* = 0.697; Figure 5F). However, when segregating tissue samples between inflamed and non-inflamed mucosa, opposite effects were observed depending of the inflammatory status of tissue: a positive, though non-significant correlation of *TFRC* expression and systemic serum free iron in non-inflamed tissue samples (Figure 5G, *r* = 0.194 and *p* = 0.119), whereas inflamed tissue presented a significant inverse correlation between *TFRC* and serum free iron levels (Figure 5H; *r* = −0.434 and *p* < 0.01). These results illustrate an inflammation-driven transcriptional regulation of mucosal *TFRC* gene expression, where, especially in the inflamed mucosa, serum free iron levels dictate the expression of *TFRC*, which was highest when systemic free iron levels were decreased in these patients and it is likely regulated by HIF1α pathway activation.

### HIF1α Pathway Activation Regulates Gene Expression of *TFRC* in Intestinal Epithelial Cells *In Vitro*

**FIGURE 6:**
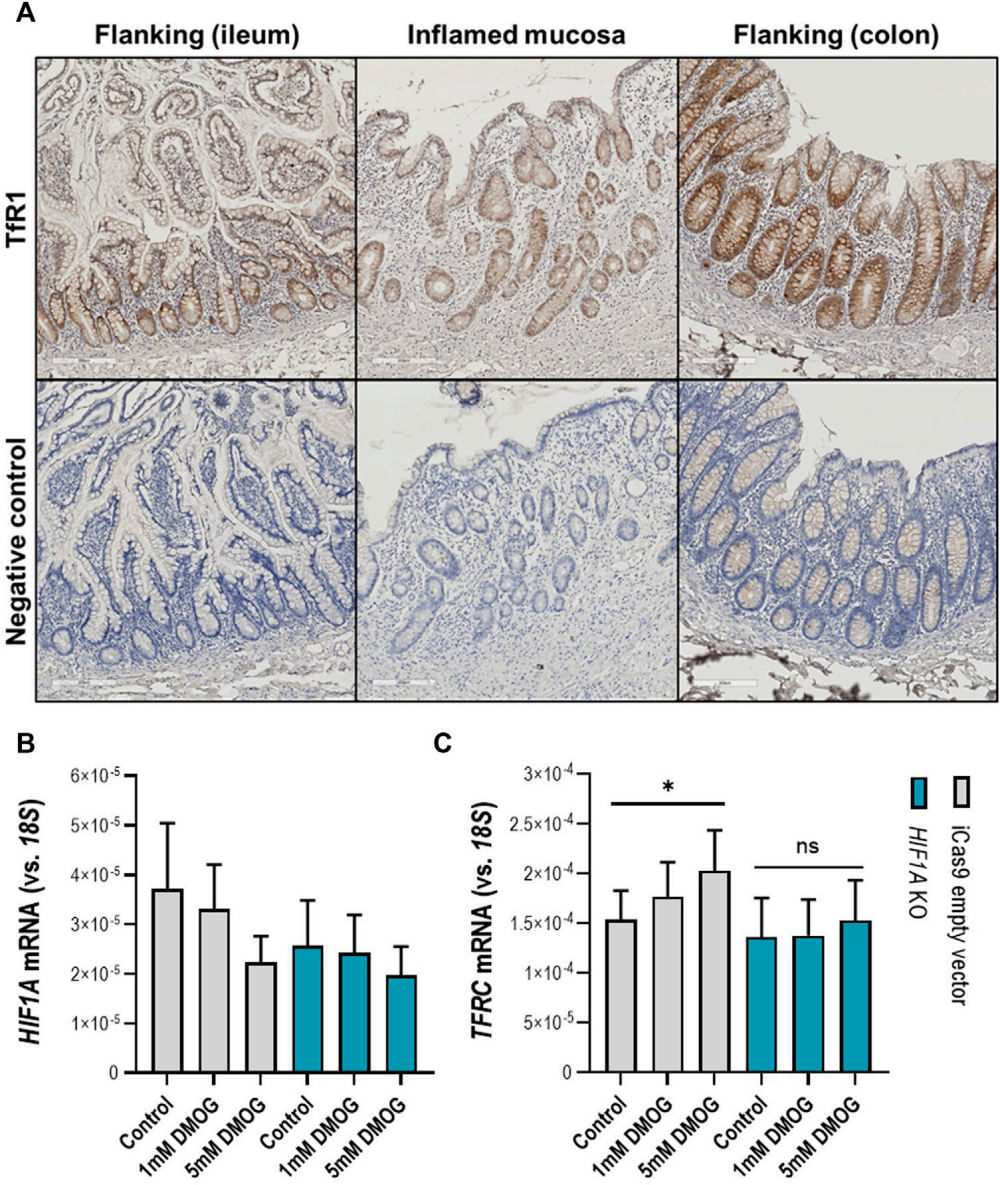
**(A–C)** *TFRC* gene expression in human intestinal epithelial cells. **(A)** Transferrin receptor protein-1 (TfR1) immunohistochemistry staining in inflamed (ileocecal) and flanking regions of the human intestinal mucosa shows enrichment of TfR1 protein levels in the epithelial layer. Respective negative control staining is presented in the panel below. Control and stable *HIF1A*-null Caco-2 cells treated with DMOG in two concentrations and the effect on expression levels of **(B)** *HIF1A* and **(C)** *TFRC*. All data are presented as mean ± SEM. DMOG, Dimethyloxallyl Glycine. All the conditions were performed in duplicate to a *N* = 3; ^*^ *p* < 0.05; ns, not significant.

Following the observation that in the inflamed ileal mucosa *TFRC* expression is at the highest levels compared to non-inflamed mucosa (Figure 5E), we aimed to examinate whether activation of the HIF1α pathway in physiological (non-inflamed) conditions can induce *TFRC* expression in intestinal epithelial cells. Firstly, we examined the expression pattern of TfR1 (protein encoded by *TFRC*) in inflamed and flanking (ileal and colonic) mucosa of IBD patients (Figure 6A). We observed an enrichment for TfR1 in the epithelial layer of both inflamed and non-inflamed mucosa (upper panel on Figure 6A), especially in crypts. Next, we treated intestinal epithelial Caco-2 cells with the hydroxylase inhibitor and HIF pathway agonist DMOG (1 and 5 mM; Figure 6B, C). DMOG treatment did not affect expression levels of the intact HIF1A gene in Caco-2 control cells (Figure 6B, grey bars), nor of the inactivated HIF1A gene in Caco-2-HIF1A-null cells in which HIF1A was stably inactivated by CRISPR/Cas9 technology (Figure 6B, blue bars). However, DMOG dose-dependently induced TFRC expression [Figure 6C, grey bars (*p* < 0.05)], which was blunted in similarly-treated Caco-2-HIF1A-null cells (blue bars in Figure 6B, C). This demonstrates that TFRC gene expression levels are transcriptionally regulated by HIF1α in noninflamed intestinal epithelial cells.

## Discussion

In this study, we show a location-specific mucosal transcriptional response to inflammation and iron deficiency of HIF1α-target genes (particularly of *TFRC*) in intestinal mucosa of patients with IBD. We show a transcriptional signature of the intestinal HIF1α pathway that is primarily driven by anatomical location and strongly altered by intestinal inflammation. In this respect, ileal tissue showed a higher HIF1α activation capacity (calculated as HIF scores) than colonic tissue. Moreover, we described that HIF1α targets were highly upregulated in inflamed ileal mucosa, compared to non-inflamed ileal tissue, especially the *TFRC* gene encoding transferrin receptor protein 1 (TFR1), which is the major iron uptake transmembrane protein. Furthermore, we show that *in vitro* activation of HIF1α by the HIF-hydroxylase inhibitor DMOG leads to upregulation of *TFRC* expression in Caco-2 cells grown in physiological/non-inflamed conditions, which is dependent on HIF1α. Taken together, we described a pathophysiological phenomenon of regulation of gene expression of the iron transporter TFR1 that takes place in the inflamed ileal mucosa and is exacerbated by systemic iron deficiency, which sheds light on a potential crosstalk with HIF1α pathway activation, that can still be activated in non-inflamed circumstances. This, in turn, may be amenable to therapeutic intervention (e.g., by HIF agonists such as DMOG) at both the inflamed and non-inflamed intestinal mucosa in order to promote intestinal iron uptake and prevent iron deficiency.

The transcriptional regulation of HIF1α depends on the co-expression of its negative regulators, which varies strongly between different tissues. Thoroughly revised by Taylor and Colgan (2017), different cell types tolerate niche-specific oxygen levels throughout the body, setting a different threshold depending on the tissue of origin (Taylor and Colgan, 2017). This phenomenon is of special importance to the intestinal mucosa, which encounters a state of hypoxia even in physiological conditions (Fagundes and Taylor, 2017; Brown et al., 2020). Interestingly, here we demonstrate that the anatomical location in the intestine determines the activation pattern of HIF pathways in the intestinal mucosa, where ileal tissue presents a higher HIF1α activation capacity (calculated as HIF1α score) compared to colonic tissue. However, we found that expression levels of HIF-target genes are, in general, higher in colonic than in ileal tissue. Although this may seem contradicting, we observed that expression levels of the asparaginyl-hydroxylase FIH (encoded by the *HIF1AN* gene) are higher in ileal tissue, compared to colonic tissue. We therefore hypothesize that this negative regulator of HIF plays a substantial role in the HIF-mediated gene expression in ileal mucosa. A possible explanation for the higher levels of HIF target genes in colonic tissue is that mucosal oxygen levels are lower in the colon compared to the small intestine (He et al., 1999; Fisher et al., 2013; Singhal and Shah, 2020). As such, ileal HIF-pathway activation may be more dynamically regulated compared to the more equal anaerobic colonic tissue microenvironment. However, our study design did not include O_2_ partial pressure measurements at the mucosal sites, which could be valuable to further elucidate the possible impact of oxygen gradient steepness. Moreover, because of the post-translational nature of HIF1α (and HIF2α) regulation, our RNA-seq findings could be improved by studying the protein levels of both HIF1α and HIF2α at the intestinal mucosa of patients with IBD. Thus, further research is needed to better understand both phenomena.

TFR1, encoded by *TFRC*, facilitates clathrin-mediated endocytosis of transferrin-bound iron, after which Fe^3+^ iron is released and transported into the cytoplasm by divalent metal transporter 1 (DMT1). Subsequently, the transferrin-TfR1 complex is recycled back to the cell membrane (Anderson et al., 2012). In hypoxic conditions, HIF1α has been shown to bind to the promotor region of *TFRC*, positively regulating its expression in hypoxia in various (non-intestinal) human cell lines (Lok and Ponka, 1999; Tacchini et al., 1999; Xu et al., 2017). The stability of *TFRC* mRNA is also under post-transcriptional control by iron and iron-regulatory proteins (IRP) that can bind to iron-responsive elements (IREs) at the 3′-untranslated region of *TFRC* mRNA, although stimulation of *TFRC* expression upon hypoxia is primarily of transcriptional origin (Tacchini et al., 1999). We demonstrated that HIF1α also promotes *TFRC* expression in human intestinal epithelial cells. Furthermore, it is important to notice that *TFRC* expression is regulated by the hepatic hormone hepcidin, a master regulator of iron absorption and systemic iron homeostasis, being upregulated when inflammatory cytokines accumulate in the circulation (Nemeth et al., 2004; Murawska et al., 2016). Hepcidin suppresses cytosolic iron export and limits the efflux of iron into the circulation (Brissot et al., 2018). Interestingly, hepcidin expression is suppressed by hypoxia, in a mechanism controlled by HIF2α, which stimulates iron absorption (Mastrogiannaki et al., 2009; Shah et al., 2009; Xu et al., 2017; Schwartz et al., 2019). We hypothesize that low serum iron levels trigger the induction of *TFRC* expression in the intestinal mucosa *via* HIF1α. Transferrin-bound iron sequestration from the gut lumen across the intestinal mucosa has been described (Huebers et al., 1983; Kolachala et al., 2007), for which this TFR1-HIF1α crosstalk may improve oral iron absorption. Furthermore, both low systemic iron levels and pathophysiologic hypoxia suppress hepcidin expression, thereby increasing intracellular iron mobilization. Furthermore, HIF-hydroxylase inhibitors, such as DMOG, are also known to activate the HIF2α pathway, which may potentially have therapeutic applications to recover IBD patients from anemia, *via* downregulation of hepatic hepcidin expression. Finally, we have demonstrated an enrichment of *TFRC* expression in the intestinal epithelium of both inflamed and non-inflamed intestinal mucosa, which may suggest that the regulation of the *TFRC*-HIF1α axis is predominantly epithelial in nature. However, recent studies examining *TFRC* regulation in pulmonary inflammation have shown that *TFRC* expression was associated with infiltrating immune cells, such as macrophages (Khadem Ali et al., 2020). In this context, one may hypothesize that iron sequestration is influenced by factors such as host-microbial competition for iron and oxidative stress. Considering these findings, future research may focus on the potential contribution of immune cells in the expression of *TFRC*-HIF1α-related genes.

**FIGURE 7:**
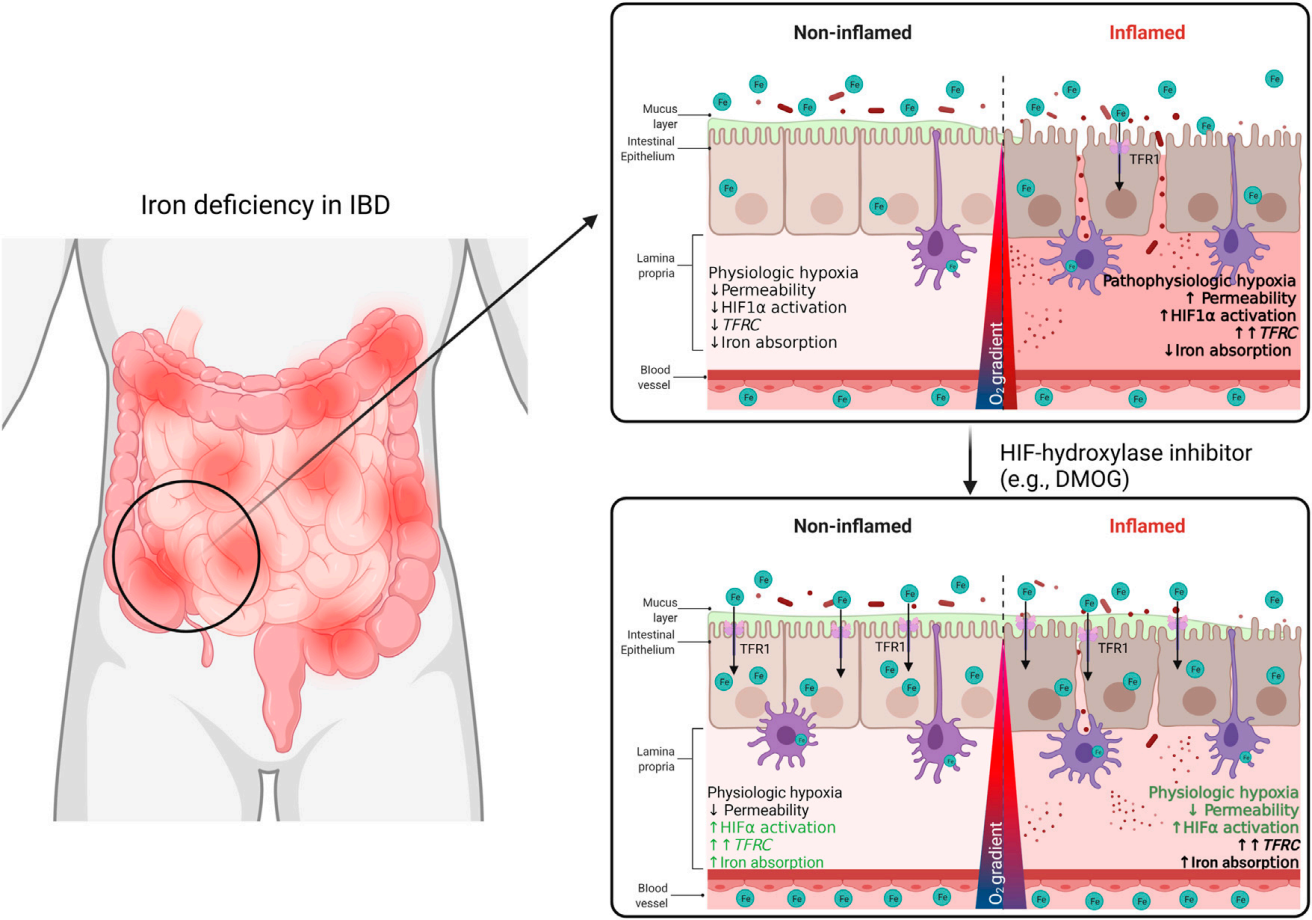
In this study, we demonstrate a TFR1-HIF1α axis may be a target of interest for the regulation of systemic iron homeostasis in the context of IBD. We describe a correlation between *TFRC* expression and HIF1α pathway activation in the intestinal mucosa of IBD patients. This *TFRC*-HIF1α axis is regulated by inflammation and systemic iron deficiency. Patients’ responsiveness to iron supplementation may be improved with treatment with HIF1α-agonists.

The therapeutic potential of HIF-hydroxylase inhibitors is currently being evaluated in phase 3 clinical trials (Haase, 2017). These are mainly focused on the HIF-mediated induction of erythropoietin in patients with chronic renal anemia. Examples of promising HIF-hydroxylase inhibitors are GKS-1278863, FG-4592, and AKB-6548, all of which increase circulating erythropoietin and help to alleviate anemia in these patients [reviewed by Haase (2017)]. Moreover, pre-clinical studies have shown that HIF-hydroxylase inhibitors, such as DMOG, ameliorate experimental colitis in a variety of animal models (Colgan et al., 2020). We show that the inflamed ileal mucosa in IBD patients has an increased HIF1α score and is more sensitive to variations in systemic iron levels. This crosstalk is reflected on the fine-tuning of the expression of *TFRC* in the inflamed intestinal mucosa, which is not observed in non-inflamed mucosa. In line with this observation, we hypothesize that treatment with HIF-hydroxylase inhibitors (i.e., HIF agonist) is potentially beneficial for IBD patients by exerting a dual effect: 1) suppression of mucosal inflammation, as demonstrated in *in vivo* studies (Cummins et al., 2008; Robinson et al., 2008; Tambuwala et al., 2015; Colgan et al., 2020), and 2) ameliorate iron deficiency and anemia by stimulating intestinal iron absorption in the non-inflamed mucosa (our study), in combination with systemic endocrine effects (e.g., upregulation of erythropoietin) (Figure 7). *Via* the latter mechanism, activation of the HIF pathway aimed to increase *TFRC* expression in non-inflamed tissue regions may be of particular therapeutic value, as in inflamed tissue this activation is already established and likely cannot be further enhanced.

To the best of our knowledge, our study is the first to show an association between ID in IBD patients and activation of HIF1α target genes, which may pave the way towards novel therapeutic targets in future clinical studies. However, several limitations of our study also need to be considered. First, this study was of retrospective, cross-sectional, associative nature and did not include patient follow-up, therapeutic effects or validation in an independent patient cohort. Furthermore, availability of laboratory data closest to biopsy collection date represented a limitation to our group sizes. To partially overcome this limitation, we restricted ourselves to the mechanistic discoveries in this study and strengthened this with an *in vitro* validation of one of our main findings using human intestinal epithelial cells. Second, the limited (sub)group sizes and disease heterogeneity in this cohort did not permit to reliably adjust for potential confounding factors or to allow further patient stratification. Finally, we observed a significant difference in corticosteroid use in ID patients compared to non-ID patients in our cohort, which could influence the mucosal HIF1α response. For example, a bi-directional effect between glucocorticoids and HIF1α activation in different cell types has been extensively reviewed elsewhere (Vanderhaeghen et al., 2021). However, in our study, comparative analyses for gene expression were all adjusted for medication use in linear mixed models and steroid use did not have a significant impact on intestinal mucosal gene expression. Furthermore, we estimate that it is unlikely that use of steroids significantly affected our results (Figure 3A).

In conclusion, our results demonstrate that intestinal location and inflammation are the major determinants of HIF1α pathway expression and activation in the intestinal mucosa of patients with IBD. Inflammation and systemic iron deficiency cumulatively enhance gene expression of ileal mucosal *HIF1A* and *TFRC*, where circulating free iron negatively associates with *TFRC* expression. Finally, *TFRC* expression is induced by HIF-hydroxylase inhibition in Caco-2 cells in a HIF1α-dependent manner, suggesting the TFR1-HIF1α axis is a target of interest for the regulation of systemic iron homeostasis. The effect of HIF-agonists in inflamed and non-inflamed intestinal may increase patients’ responsiveness to either oral or intravenous iron supplementation and diminish intestinal mucosal inflammation.

## Data Availability

The datasets presented in this study can be found in online repositories. The names of the repository/repositories and accession number(s) can be found below: https://www.ebi.ac.uk/ena, EGAS00001002702.
