# Supplementary material for: HIF1α-Dependent Induction of TFRC by a Combination of Intestinal Inflammation and Systemic Iron Deficiency in Inflammatory Bowel Disease
Source: Front Physiol. 2022 Jun 8;13:889091. doi: 10.3389/fphys.2022.889091 (PMC9214203; doi:10.3389/fphys.2022.889091)
Supplement: Supplementary file 1 [file Table1.DOCX]

Supplementary Material

# Supplementary Figures and Tables

## Supplementary Figures


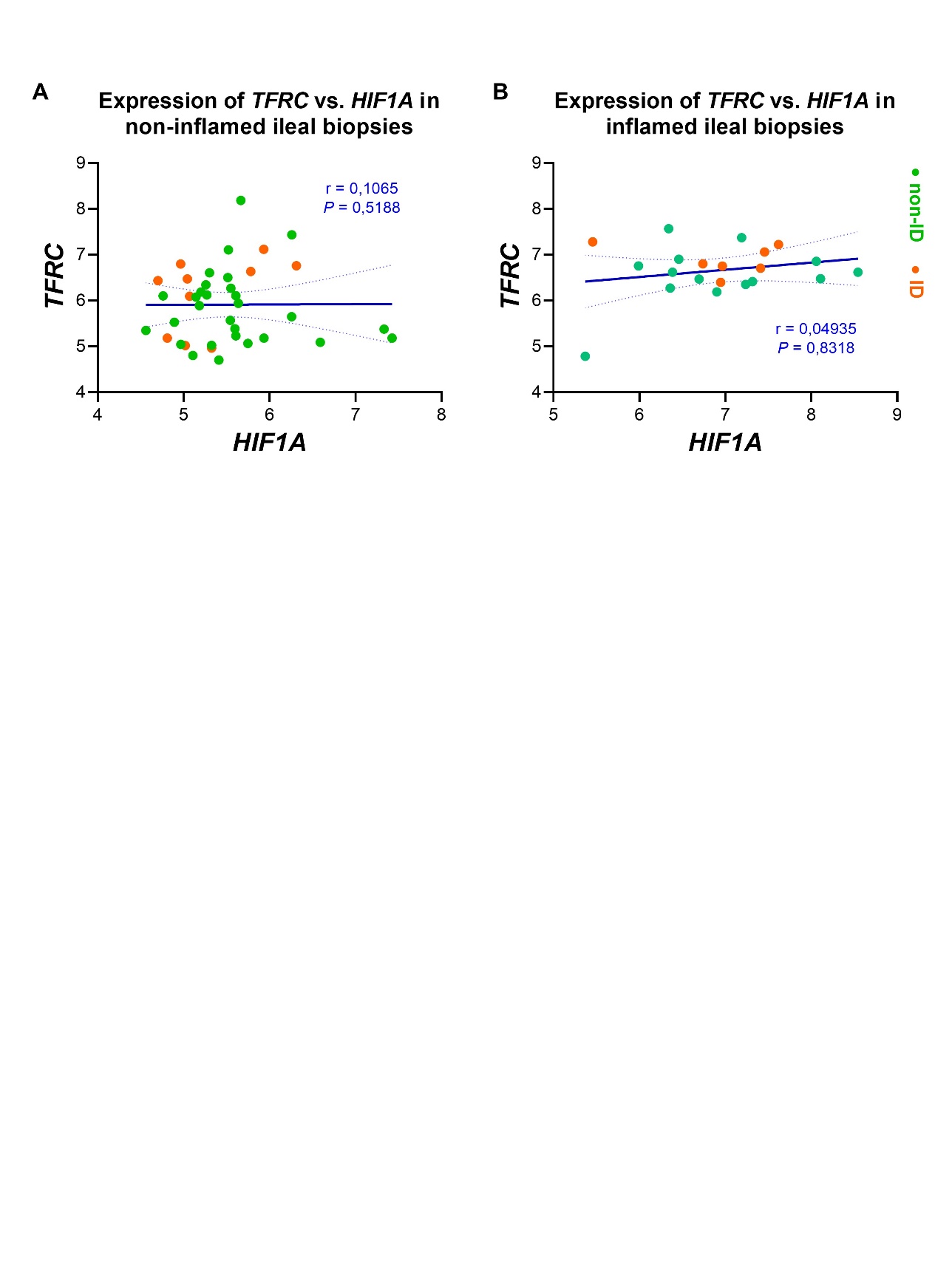


**Supplementary Figure S1.** Spearman correlation analysis between gene expression levels of *TFRC* and *HIF1A* in ileal mucosa from (**A**) non-inflamed and (**B**) inflamed areas. Samples presented as orange dots are from IBD patients with ID, while green dots represent samples from IBD patients with normal iron levels (non-ID). Data presented in correlation graphs are presented as best fitting line with 95% confidence intervals.

## Supplementary Tables

| **Supplementary Table S1.** Descriptive statistics of IBD patients with differences shown between IBD patients with normal iron status (non-ID) and iron deficiency (ID). | | | |
| --- | --- | --- | --- |
| **Variable** | **CD** | **UC** | ***P*-value** |
|  | *n* = 61 | *n* = 34 |  |
| Age (years) | 43.3 ± 13.6 | 46.0 ± 16.4 | 0.411 |
| Female gender, *n* (%) | 39 (63.9) | 15 (44.1) | 0.062 |
| BMI (kg/m^2^) | 24.9 ± 4.0 | 26.0 ± 4.5 | 0.218 |
| Current smoking, *n* (%) | 13 (21.3) | 6 (17.6) | 0.669 |
| *Iron Status* |  |  | 0.206 |
| Non-ID, *n* (%) | 41 (67.2) | 27 (79.4) |  |
| ID, *n* (%) | 20 (32.8) | 7 (20.6) |  |
| *Montreal classification (Age)* |  |  |  |
| A1 (≤ 16 years), *n* (%) | 5 (8.2) | 5 (15.2) |  |
| A2 (17 – 40 years), *n* (%) | 42 (68.9) | 15 (45.5) |  |
| A3 (> 40 years), *n* (%) | 14 (23.0) | 13 (39.4) |  |
| *Montreal classification (Location) (CD)* |  |  |  |
| L1 (ileal disease), *n* (%) | 15 (24.6) | - |  |
| L2 (colonic disease), *n* (%) | 12 (19.7) | - |  |
| L3 (ileocolonic disease), *n* (%) | 27 (44.3) | - |  |
| L4 (upper GI disease), *n* (%) | 1 (1.6) | - |  |
| *Montreal classification (Behavior) (CD)* |  |  |  |
| B1 (non-stricturing, non-penetrating), *n* (%) | 28 (45.9) | - |  |
| B2 (stricturing), *n* (%) | 24 (39.3) | - |  |
| B3 (penetrating), *n* (%) | 9 (14.8) | - |  |
| *Montreal classification (Perianal disease) (CD)* |  |  |  |
| Yes, *n* (%) | 21 (34.4) | - |  |
| No, *n* (%) | 40 (65.6) | - |  |
| *Montreal classification (Extension) (UC)* |  |  |  |
| E1 (proctitis), *n* (%) |  | 2 (6.1) |  |
| E2 (left-sided colitis), *n* (%) |  | 11 (33.3) |  |
| E3 (pancolitis), *n* (%) |  | 20 (60.6) |  |
| *Montreal classification (Severity) (UC)* |  |  |  |
| S0 (remission), *n* (%) |  | 1 (3.0) |  |
| S1 (mild disease), *n* (%) |  | 5 (15.2) |  |
| S2 (moderate disease), *n* (%) |  | 18 (54.5) |  |
| S3 (severe disease), *n* (%) |  | 9 (27.3) |  |
| *Concomitant medication use (IBD)* |  |  |  |
| Medication (any), *n* (%) | 49 (80.3) | 33 (97.1) |  |
| Aminosalicylates, *n* (%) | 4 (6.6) | 30 (88.2) |  |
| Thiopurines, *n* (%) | 21 (34.4) | 10 (29.4) |  |
| Corticosteroids, *n* (%) | 21 (34.4) | 11 (32.4) |  |
| TNF-antagonists, *n* (%) | 22 (36.1) | 4 (11.8) |  |
| Methotrexate, *n* (%) | 8 (13.1) | 0 (0.0) |  |
| Data are presented as mean ± standard deviation (SD), median [interquartile range, IQR] or proportions n with corresponding percentages (%). *P-value indicating statistical significance. | | | |

| **Supplementary Table S2.** Relevant laboratory parameters of IBD patients with differences shown between IBD patients with normal iron status (non-ID) and iron deficiency (ID). | | | |
| --- | --- | --- | --- |
| **Variable** | **CD** | **UC** | ***P*-value** |
|  | ***n* = 61** | ***n* = 34** |  |
| Hemoglobin (mmol/l) | 8.4 ± 0.8 | 8.5 ± 1.1 | 0.439 |
| MCV (fl) | 90.6 ± 6.7 | 89.6 ± 5.5 | 0.492 |
| CRP (mg/l) |  |  | 0.803 |
| *≤ 5 mg/l* | 48 (78.7) | 26 (76.5) |  |
| *> 5 mg/l* | 13 (21.3) | 8 (23.5) |  |
| Free iron (µmol/l) | 14.9 ± 7.5 | 16.1 ± 8.1 | 0.553 |
| Ferritin (µg/l)^†^ | 39 [23;91] | 68 [28;135] | 0.142 |
| TYBC (µmol/l) | 67.2 ± 14.5 | 61.1 ± 10.6 | 0.083 |
| Transferrin (g/l) | 2.8 ± 0.6 | 2.6 ± 0.5 | 0.291 |
| Transferrin saturation (%)^†^ | 20.0 [16.3;27.8] | 23.3 [18.0;38.5] | 0.243 |
| Data are presented as mean ± standard deviation (SD), median [interquartile range, IQR] or proportions *n* with corresponding percentages (%). *P-value indicating statistical significance. | | | |

| **Supplementary Table S3.** Sequences of probes and primers used for quantitative RT-PCR | | | |
| --- | --- | --- | --- |
| **Gene** | **Organism** | **Sequence** | |
| *18S* | Human | Probe | 5'-CGC GCA AAT TAC CCA CTC CCG A-3' |
|  |  | Sense | 5'-CGG CTA CCA CAT CCA AGG A-3' |
|  |  | Anti-sense | 5'-CCA ATT ACA GGG CCT CGA AA-3‘ |
| *TFRC* | Human | Probe | 5’-TCAAAGACAGCGCTCAAAACTCGGTGA-3’ |
|  |  | Sense | 5’-TGGCGTGATCAACATTTTGTTAA-3’ |
|  |  | Anti-sense | 5’-CCACATAACCCCCAGGATTCT-3’ |
| *HIF1A* | Human | Probe | 5-TTGCACTGCACAGGCCACATTCAC-3’ |
|  |  | Sense | 5’-TGAACATAAAGTCTGCAACATGGA-3’ |
|  |  | Anti-sense | 5’-TGAGGTTGGTTACTGTTGGTATCATATA-3’ |
