# Supplementary figures and images for: HIF1α-Dependent Induction of TFRC by a Combination of Intestinal Inflammation and Systemic Iron Deficiency in Inflammatory Bowel Disease
Source: Front Physiol. 2022 Jun 8;13:889091. doi: 10.3389/fphys.2022.889091 (PMC9214203; doi:10.3389/fphys.2022.889091)

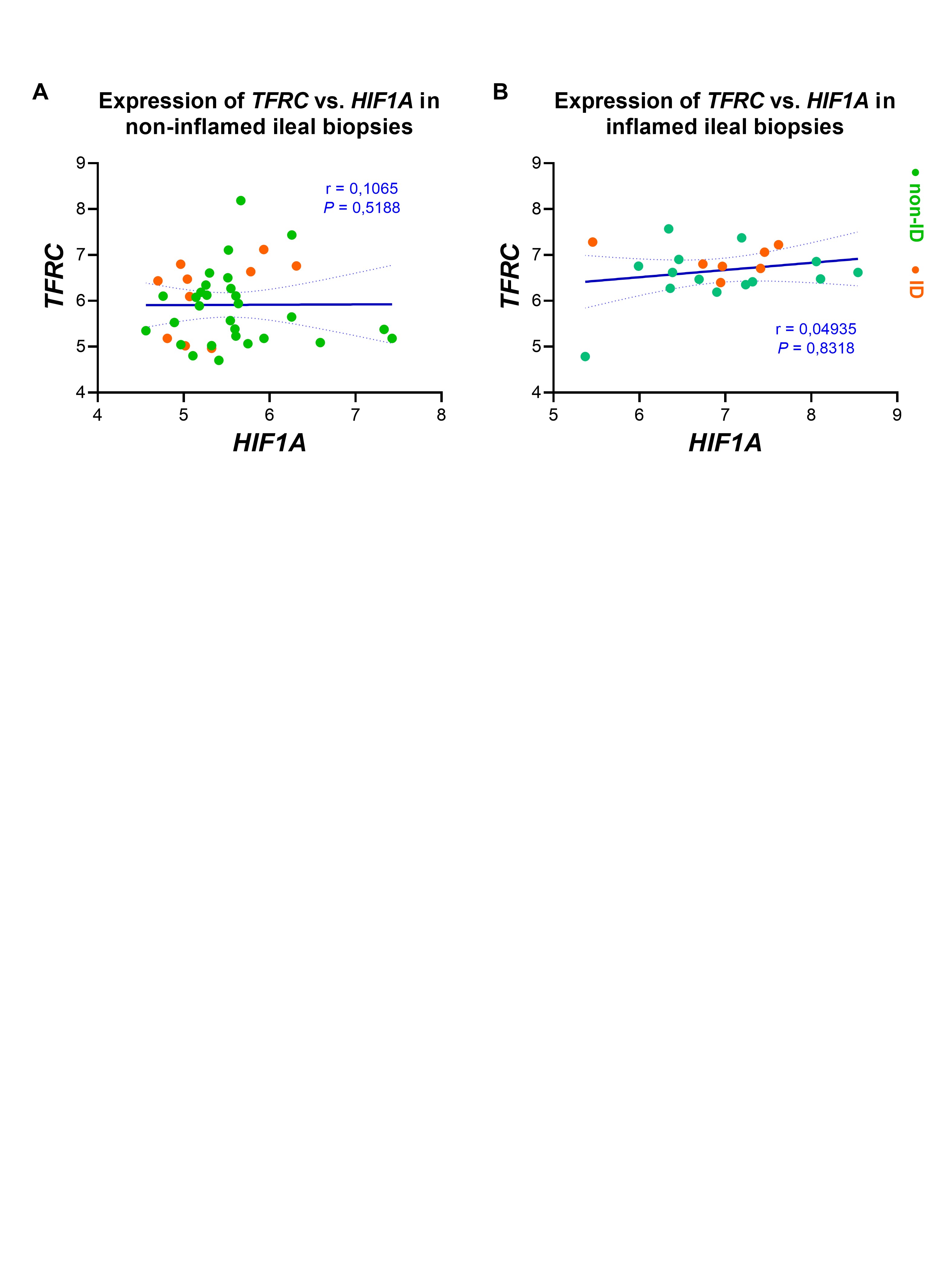

Supplement: Supplementary file 2 [file Image1.JPEG]
